# Adsorption of organic pollutants from real refinery wastewater on prepared cross-linked starch by epichlorohydrin

**DOI:** 10.1016/j.dib.2018.05.060

**Published:** 2018-05-18

**Authors:** Riyadh K. Abid, Ammar S. Abbas

**Affiliations:** a Petroleum Research Center, Ministry of Science and Technology, Iraq; b Chemical Engineering Department – College of Engineering – University of Baghdad, Iraq

**Keywords:** Adsorption, Organic pollutants, Wastewater, CLS, Epichlorohydrin

## Abstract

The presented data shows how cornstarch can be modified to a material that can effectively remove organics pollutants from a real wastewater. A low-cost adsorbent obtained from cornstarch by the crosslinking with epichlorohydrin. The prepared crossed linked starch (CLS) adsorbent characterized by X-ray diffraction, zeta potential, Fourier-transform infrared spectroscopy, atomic force microscopy, scanning electron microscopy, and Brunauer–Emmett–Teller surface area. The effects of initial chemical oxygen demand of the real wastewater, temperature and time of the adsorption of the organics on the prepared CLS were analyzed. The removal of the highest organics concentration was 89.85%. Langmuir and Freundlich isotherm models have been applied to investigate the adsorption equilibrium. The maximum adsorption capacity of the organics pollutants on the prepared CLS was 256.41 mg/g. Thermodynamic parameters show that the adsorption process of organics on CLS is more favorable at low temperature.

## Specifications Table

**Table:** 

Subject area	*Chemical Engineering*
More specific subject area	*Adsorbent preparation and adsorption*
Type of data	*Figure and tables*
How data was acquired	by X-ray diffraction (XRD), zeta potential, Fourier-transform infrared spectroscopy (FTIR), atomic force microscopy (AFM), scanning electron microscopy (SEM), and Brunauer–Emmett–Teller (BET) surface area
Data format	*Analyzed*
Experimental factors	*The prepared CLS was characterized and performed by its ability to remove organics from real untreated wastewater rejected from Dora Refinery in Baghdad (Iraq)*
Experimental features	*Preparation and characterization of CLS and its performance for the removal of organics pollutant by adsorption*
Data source location	*University of Baghdad and Petroleum Research Center*
Data accessibility	*Data are accessible with this article*

## Value of the data

• The prepared CLS adsorbent has a worthy prospective application related to real wastewater treatment.
• The characterization data of the CLS are useful for the scientific community to promote explorations of environmental-friend adsorbents.
• The data of isotherms, kinetics, and thermodynamics will be informative for predicting and modeling of the adsorption of organics from wastewater by CLS.

## 1 Data

**Fig. 1:**
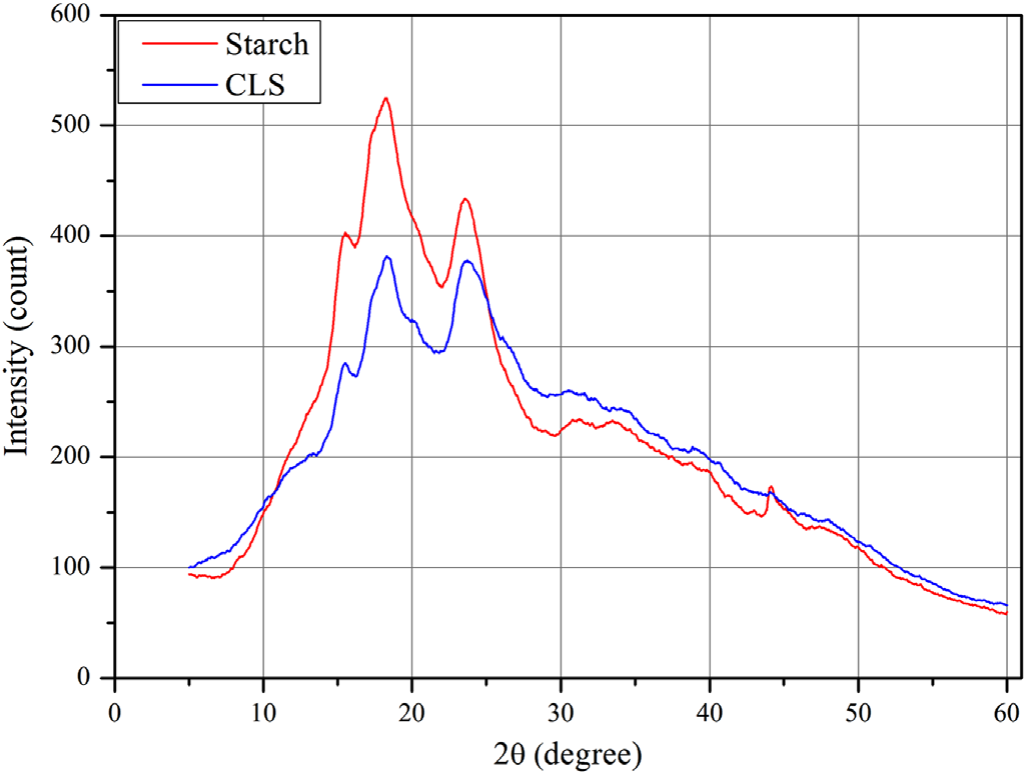
X-ray diffractograms of starch and CLS.

**Fig. 2:**
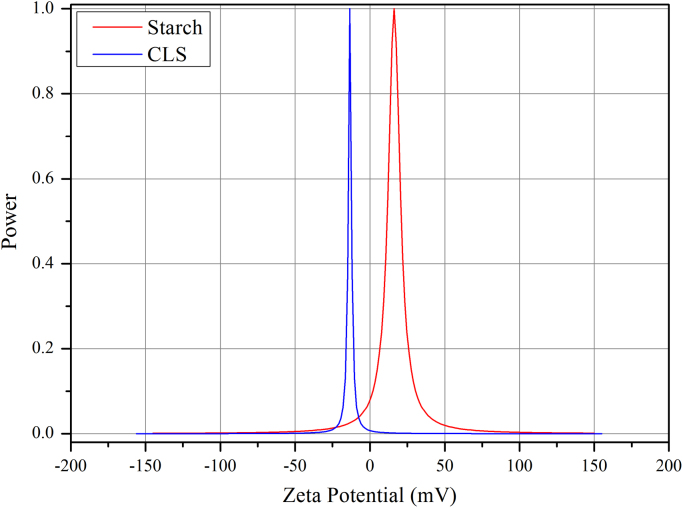
Zeta potential for starch and CLS.

**Fig. 3:**
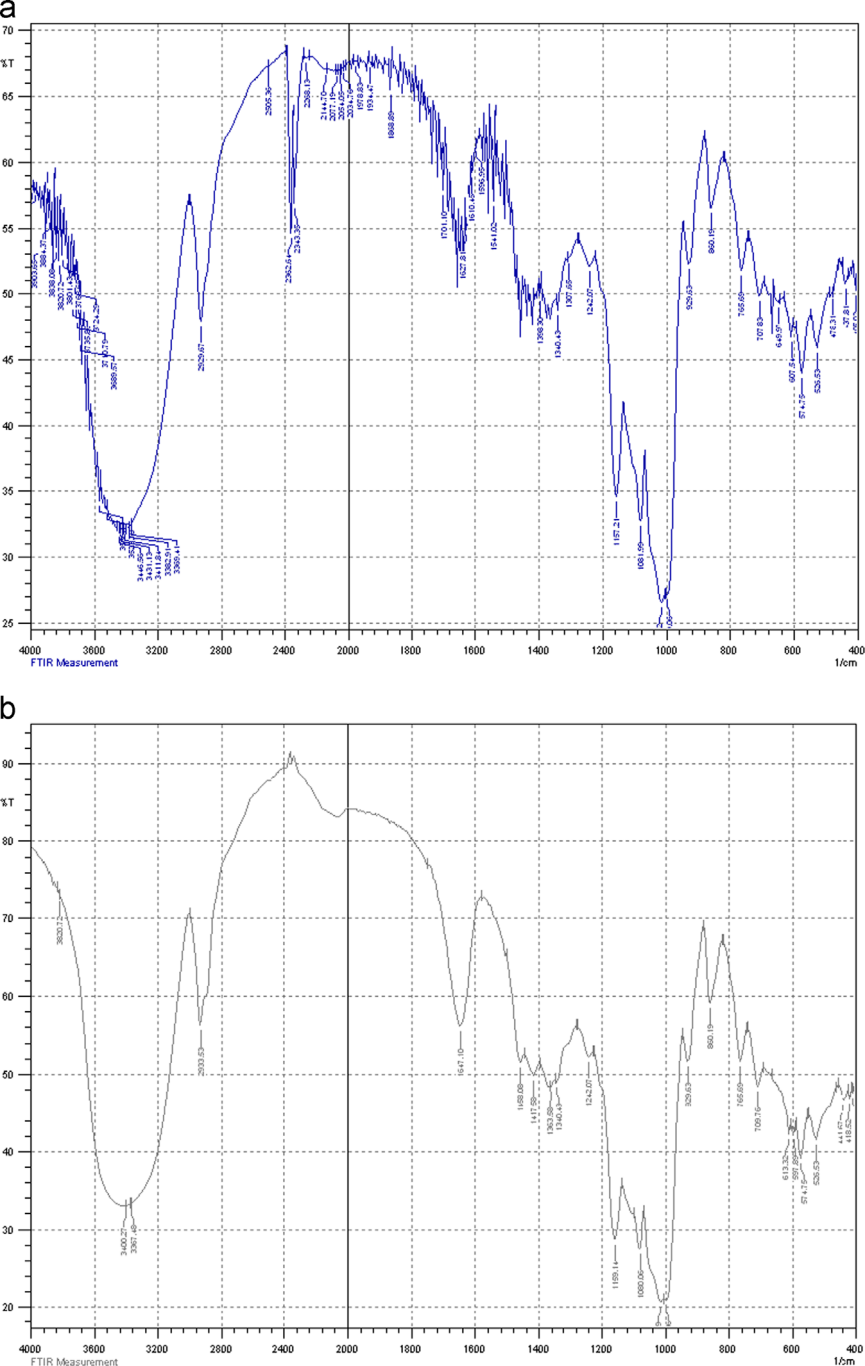
FTIR test for (a) starch and (b) CLS.

**Fig. 4:**
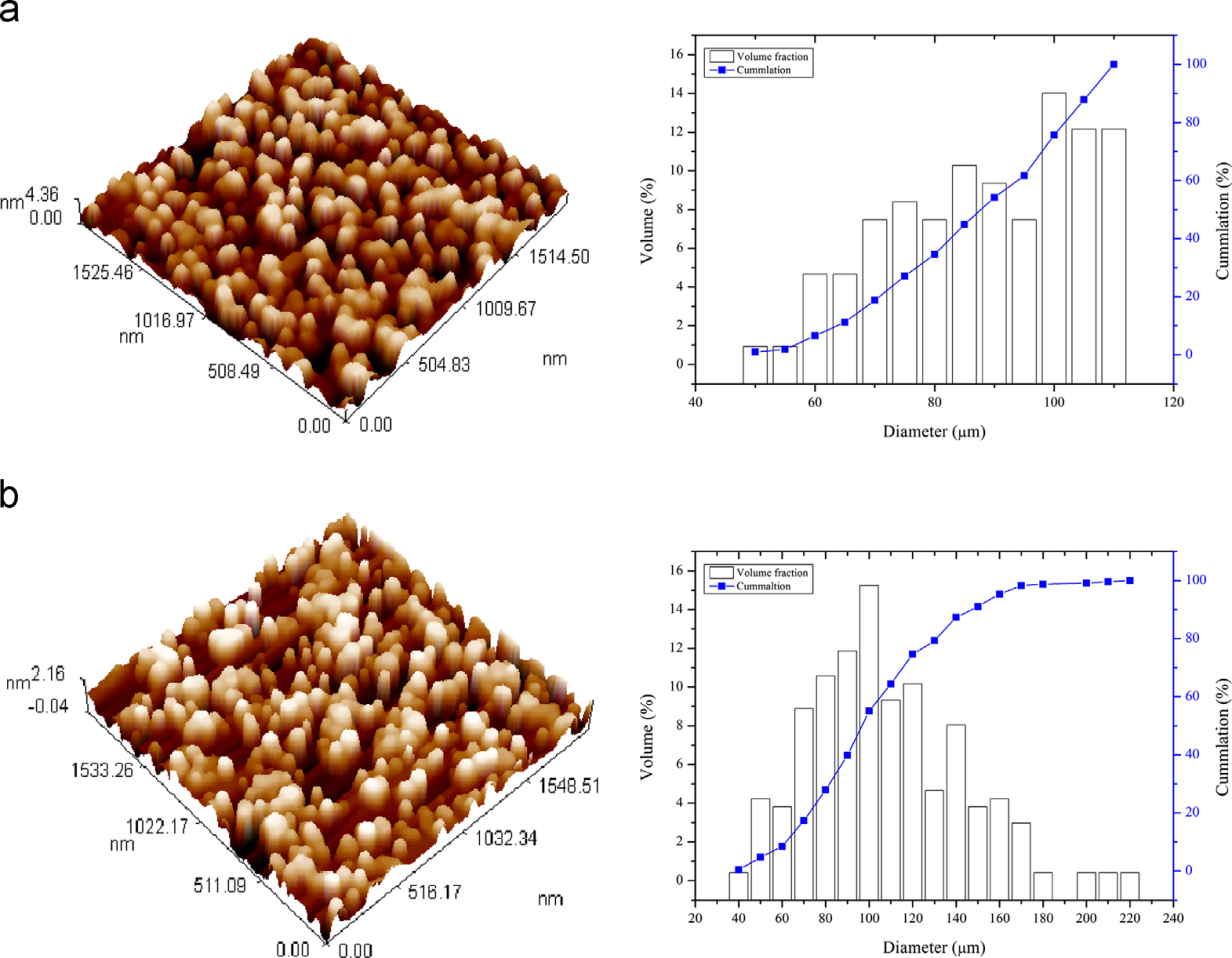
AFM and particles distribution for (a) starch and (b) CLS.

**Fig. 5:**
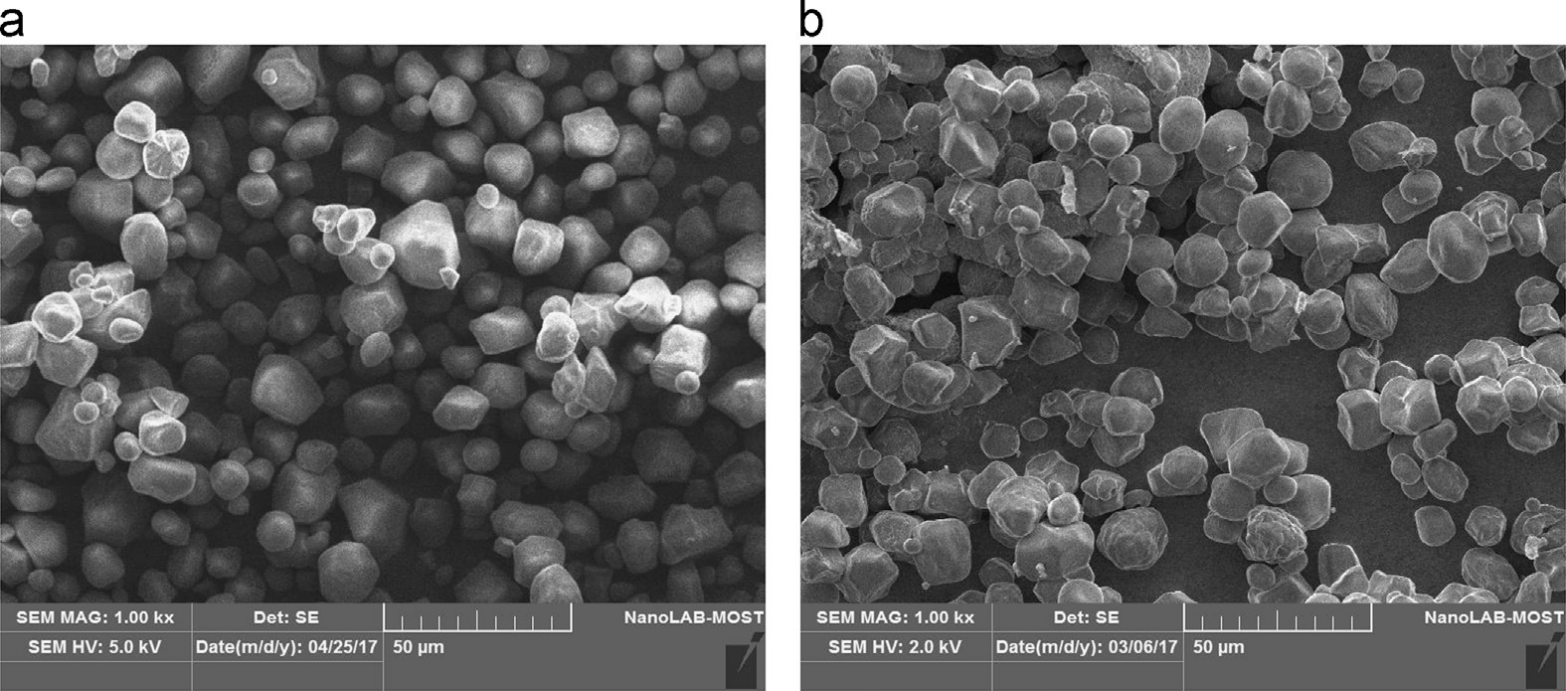
SEM for (a) starch (×1000) and (b) CLS (×1000).

The XRD of the native corn starch and promising modified starch are given in Fig. 1 using Shimadzu X-ray 6000. The zeta potential analyses achieved using Brookhaven instrument and Fig. 2 consists of these tests. Decreasing in the intensity of O–H bonds indicates crosslinking process using FTIR as shown in Fig. 3. The images outlines from AFM and SEM for both types of starch also illustrated in Fig. 4, Fig. 5. The pH of the solutions was measured. Constants for different isotherm models presented in Table 1. The adsorption rate constants for both models (Langmuir and Freundlich) at the various temperatures shown in Table 2. Pseudo-first and second-order kinetic models, isotherms and thermodynamic parameters were analyzed using Langmuir and Freundlich models. Adsorption experiments were carried out by the batch method. A dose of one gram of prepared CLS was added to each liter of untreated wastewater stream rejected (and its diluted solutions) from Dora Oil Refinery – Baghdad. The chemical oxygen demand (COD) of the rejected wastewater was 362 mg/l and pH was around 6.8–7.

**Table 1:** Constants for different isotherm models.

Isotherm model	Model parameters	Parameter value	*R*^2^
Langmuir	$q_{m}$, mg/g	256.41	0.9673
	$K_{L}$, l/mg	0.052	
Freundlich	$K_{F}$, (mg/g)/(mg/l)^1/n^	11.788	0.9549
	*n*, –	1.195

**Table 2:** Rate constant values for adsorption of organics on CLS.

Adsorption kinetic model	Model constants	Model parameter value at temperature		
		25 °C	40 °C	60 °C
Pseudo-first-order	$k_{1}$, (1/min)	0.1018	0.0467	0.0398
	$q_{e}$, (mg/g)	97.71	90.38	85.29
	*R*^2^	0.8239	0.6407	0.7001
Pseudo-second-order	$k_{2}$, (g/mg min)	0.268	0.215	0.174
	$q_{e}$, (mg/g)	243.90	232.56	217.39
	*R*^2^	0.9954	0.9941	0.9844

## 2 Experimental design, materials and methods

### 2.1 Preparation and characteristics of CLS

A specified quantity of deionized water gradually added to 100 g of pre-heated cornstarch with mixing until reaching homogeneity with adding sodium chloride and sodium hydroxide solution wisely. It is an important to adjust the temperature and mixing speed through adding epichlorohydrin (EPI) as a crosslinking agent, preferably temperatures 30–35 °C. After the end of CLS preparation, a microfiltration with 0.45 µm nylon membrane and drying processes at a temperature of 50 °C is mandatory.

The majority of elemental analysis of the modified starch shown in X-ray diffractograms of pure cornstarch and promising formulation presented in Fig. 1. The most remarkable highlighted that characteristic sharp intensity diffraction peaks at 2*θ* values of 10°, 11°, 12°, 16.5°, 18°, 19.5°, 21.8°, 23°, 26.5°, 29° and 43° which reflect the crystalline nature of starch.

The zeta potential test gives an idea about an adding EPI as a cross-linked agent has a good effect as illustrated in Fig. 2; by reducing the value of zeta potential from 16.22 mV for native starch with a positive sign because of its pH value (about 3.3) to the 11.55 mV for CLS with negative sign due to its pH value (more than 5.5 to 6).

The FTIR of cross-linked starch corresponds to the superimposition of the starch with no significant shift in the major peaks but there is a decreasing in the intensity of O–H bonds at the interval around wavelength about 3200–3600 cm^−1^ which refer to crosslinking reaction occurs as demonstrated in Fig. 3.

A crosslinking is clear through reduces swelling particle of starch when affected by water solution. Fig. 4, Fig. 5 below shows the difference between native starch and cross-linked starch by AFM and SEM techniques. In AFM test (Fig. 4, a and b), the average diameter was 100.95 nm for modified starch comparing with 85 nm for native starch. Also, it was observed in CLS that repeating units of crystalline and amorphous lamellae are grouped into discrete, and the polysaccharides are arranged in concentric rings, called growth rings, irradiated from a central hilum. For SEM (Fig. 5, a and b) the shape of CLS granules became more flattening than origin starch and this analysis confirmed structure modification on the cross-linked product. The obtained BET surface area of the CLS was 0.392 m^2^/g with a pore volume of 0.000927 cm^3^/g.

### 2.2 Isotherms of organics adsorption on CLS

The amount of organics adsorbed per weight of CLS adsorbent (adsorption capacity) at equilibrium (q_e_, mg/g) was calculated from Eq. (1). Moreover, the efficiency of organics removal (EOR) was calculated from Eq. (2).

(1) $${\text{q}}_{\text{e}}\text{=}\;\frac{\text{V}\text{×}\;\left({\text{C}}_{\text{o}}-{\text{C}}_{\text{e}}\right)}{\text{m}}$$

(2) $$\mathrm{EOR}\;(\%)=\frac{C_{o}-C_{e}}{C_{o}}\times 100$$

Where *C_o_* and *C_e_* (mg/l) refer to initial and terminal (equilibrium) concentrations of organics in the adsorption solution as COD concentration, respectively, also *V* is the volume of wastewater (adsorption solution) in liter and *m* is the weight of CLS adsorbent used in gram.

Real wastewater obtained from Dora Refinery of initial COD equal to 362 mg/l (ppm) used to examine the adsorption isotherms. Most popular two-parameter isotherms [1], namely: Langmuir [2] and Freundlich [3], were selected to describe the adsorption of organic pollutants on prepared CLS. The isotherms’ constants that summarized in Table 1 were obtained by linear regression of the linear form of Langmuir and Freundlich isotherms [1] (Eqs. (3), (4)).

(3) $$\frac{1}{q_{e}}=\left[\frac{1}{q_{m}K_{L}}\right]\frac{1}{C_{e}}+\frac{1}{q_{m}}$$

(4) $$\ln\;q_{e}=\ln\;K_{F}+\frac{1}{n}\ln\;C_{e}$$

Where $q_{m}$ is the maximum adsorption capacity, $K_{L}$ is the Langmuir isotherm constant energy or net enthalpy of adsorption, $K_{F}$ is a Freundlich constant indicative of the relative adsorption capacity of the adsorbent, and *n* is the intensity or the heterogeneity factor.

### 4.3 Kinetics of organics adsorption on CLS

**Fig. 6:**
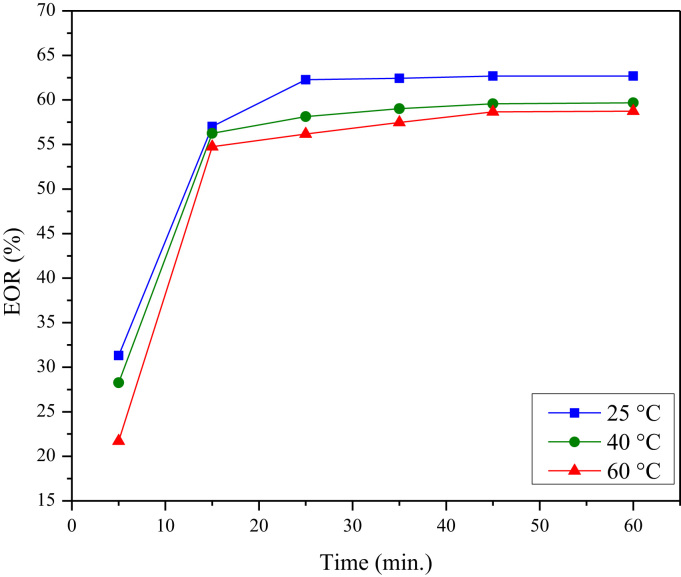
The effect of time on the organics removal at different temperatures.

The EOR was increasing sharply with the time during first 15 min and tend to unchanged after only 25 min as shown in Fig. 6.

The adsorption capacity versus time data was used to examine the best adsorption models that represented the adsorption data of organics on CLS. Kinetic models were used, namely; pseudo-first order [4] and pseudo-second-order [5]. The linear form of these models (Eqs. (5), (6)) were solved by linear regression [6] based on the least squares criterion [7] to obtain the adsorption rate constants for each model at the different temperatures as shown in Table 2.

(5) $$\mathrm{Pseudo}-\mathrm{first}\;\mathrm{order}\;\mathrm{kinetic}\;\mathrm{model:}\;\ln\left(q_{e}-q_{t}\right)=\ln q_{e}-k_{1}t$$

(6) $$\mathrm{Pseudo}-\mathrm{second}-\mathrm{order}\;\mathrm{kinetic}\;\mathrm{model:}\;\frac{t}{q_{t}}=\frac{1}{{k_{2}q}_{e}}+\frac{t}{q_{e}}$$

Where $q_{e}$ and $q_{t}$ (in mg/g) are the adsorption capacity of organics at equilibrium and at time (*t*), respectively. $k_{1}$ (1/min) and $k_{2}$ (g/mg min) are the adsorption rate constants for pseudo-first-order and pseudo-second-order kinetic model, respectively.

### 4.4 Thermodynamic of organics adsorption on CLS

Thermodynamic behavior of the organic adsorption on CLS was investigated by estimation of different thermodynamic parameters. These parameters were the change in Gibbs free energy (∆G), enthalpy (∆H) and entropy (∆S), and might found based on Eqs. (7), (8) [8]. The numerical values obtained summarized in Table 3.

(7) $$∆G=-\mathrm{RT}\;\ln(K_{d})$$

(8) ∆G=∆H−T∆S

Where *R* is the gas constant (8.314 J/mol K), and *T* (K) is the absolute temperature of the adsorption process. Though $K_{d}$ is the distribution coefficient for the adsorption of adsorbate (organics) at the adsorbent (CLS) surface and was calculated by Eq. (9).

(9) $${\text{K}}_{\text{d}}\;\text{=}\;\frac{q_{e}}{C_{e}}\left(\frac{m}{V}\right)$$

Where *q_e_* is the adsorption capacity of the adsorbent (mg/g), *C*_*e*_ (mg/l) is equilibrium concentrations of organics in the adsorption solution, *V* (l) is the volume of the adsorption solution, and *m* (g) is the weight of CLS adsorbent used.

**Table 3:** Numerical values obtained of the distribution coefficient and thermodynamic parameters versus temperature.

Temperature, K	${\text{K}}_{\text{d}}$	Thermodynamic parameters			*R*^2^
		∆G, J/mol	∆H, J/mol	∆S, J/mol K	
298	1.693	− 1304.1	− 699.95	2.02	0.9872
313	1.665	− 1326.6			
333	1.643	− 1374.5			

### 4.5 Analytical methods

Shimadzu Fourier-Transform Infrared Spectrometer apparatus used to detect the majority of elemental analysis of pure cornstarch and formulation. The specific surface area has been identified with Micromeritics Brunauer–Emmett–Teller (BET) Surface Area and Porosity Analyzer ASAP-2020. The morphological characterization of the CLS was done by (SEM, Hitachi, SU 70). The zeta potential analysis achieved using Brookhaven instrument with Smoluchowski analysis. The solutions pH analyses were performed using (inolab pH 720) meter. The COD values were obtained by the aid of Lovibond COD digestor.
